# Finite element analysis of stem migration after total hip replacement

**DOI:** 10.1007/s10237-025-01985-0

**Published:** 2025-08-18

**Authors:** Marlis Reiber, Fynn Bensel, Nils Becker, Stefan Budde, Udo Nackenhorst

**Affiliations:** 1 Institute of Mechanics and Computational Mechanics (IBNM), Leibniz University Hannover, Appelstraße 9a, 30167 Hannover, Germany; 2 TRR 298: Safety Integrated and Infection Reactive Implants (SIIRI), Hannover Medical School, Carl-Neuberg-Straße 1, 30625 Hannover, Germany; 3 International Research Training Group (IRTG) 2657, Leibniz University Hannover, Appelstraße 11/11a, 30167 Hannover, Germany; 4 Department of Orthopaedic Surgery, Diakovere Annastift, Hannover Medical School, Anna von Borries Straße 1-7, 30625 Hannover, Germany; 5 Universitätsklinikum OWL der Universität Bielefeld, Evangelisches Klinikum Bethel, Burgsteig 13, 33617 Bielefeld, Germany; 6 Chair Computational Mathematics, Institute of Mathematics, Hamburg University of Technology, Am Schwarzenberg-Campus 3, 21073 Hamburg, Germany

**Keywords:** implant stability, osseointegration, bio-active interface theory, bone-stem interface, stem subsidence

## Abstract

After total hip replacement, the primary and secondary implant stability is critical to ensure long-term success. Excessive migration of the femoral stem can cause implant loosening. In this work, a novel approach for the simulation of the femoral stem migration using the finite element method is presented. Currently, only a few mostly contact-based models exist for this purpose. Instead, a bio-active interface model is used for the bone-stem interface which transforms from the Drucker–Prager to the von Mises plasticity criterion during the osseointegration process. As the position of the implant generally stabilises within one week after the implantation, the migration and osseointegration simulations are decoupled. To understand the effects on the migration, various parameter combinations are examined and a sensitivity analysis is performed. The results indicate that the joint force and the adhesion parameter have the most substantial influence on the migration. Furthermore, the influence of the migration on the subsequent osseointegration process is explored for a numerical example. The proposed model is able to depict the femoral stem migration with values up to 0.27 mm, which are in the order of magnitude of clinically observed values. Further, the model is provided as an open-source Abaqus user material subroutine. Numerical simulation of the stem migration could assist in clinical decision-making by identifying optimal parameter combinations to improve implant stability.

## Introduction

Total hip replacement is a widely successful surgical treatment of osteoarthritis (Learmonth et al. 2007; Ferguson et al. 2018). Hip implants are divided into cemented and cementless implants. Whereas cemented implants rely on bone cement to achieve immediate primary stability, cementless implants depend on the natural development of the biomechanical properties in the bone-implant interface surrounding the femoral stem (Gao et al. 2019). Today, cementless hip implants are preferred by surgeons, and in recent years, there has been a shift from long-stem to short-stem hip implants to achieve more physiological loading and a better force distribution in the femoral bone (Learmonth et al. 2007; Floerkemeier et al. 2017).

In cementless implants, the primary stability is achieved directly after the implantation by the surgical technique. Subsequently, the secondary stability develops over several weeks or months by the bony ingrowth into the implant, a process known as osseointegration. Finally, the long-term stability refers to the overall lasting stability of the implant. Of course, the primary and secondary stability influence the long-term stability (Learmonth et al. 2007).

In general, the implant stability of cementless implants depends on several factors including the implant size, shape, coating, and position as well as the implantation procedure, e.g., the amount of press-fit and the bone quality (Floerkemeier 2021; Dittrich et al. 2019; Gao et al. 2019). Additionally, micromotions in the bone-stem interface and the size of this interface are essential for the osseointegration. However, excessive micromotions can prevent the ingrowth and may ultimately lead to the loss of the secondary stability (Gao et al. 2019).

After the implantation, every hip implant experiences some degree of stem migration. However, excessive migration can eventually lead to loosening (Krismer et al. 1999). To measure the migration, radiostereometric analysis (RSA) is often used and various RSA studies have been performed on different implant types (Floerkemeier et al. 2020; Budde et al. 2024, 2016).

Computational models can be used to assess the implant stability. The finite element method (FEM) is well established for the evaluation of the long-term stability. Thereby the bone remodelling process is simulated, a process governed by Wolff’s law, which states that bones adapt their structure to altered loading conditions (Wolff 1892). Initially, purely phenomenological models were used to study bone remodelling (Carter et al. 1989; Beaupré et al. 1990; Huiskes et al. 1992; Weinans et al. 1992; Nackenhorst 1997). Afterwards, more advanced models have been developed, incorporating features such as anisotropic behaviour and multi-scale approaches (Doblaré and García 2002; Krstin et al. 2000; Webster and Müller 2011).

Furthermore, computational models can offer valuable insights into the osseointegration and migration of cementless implant stems. These simulations can be useful for evaluating the primary and secondary stability of hip implants, as many parameters associated with the bone-implant interface cannot be directly observed (Taylor and Prendergast 2015; Gao et al. 2019). A recent literature review on finite element models for predicting aseptic loosening considering the osseointegration in the interface was presented by Sun et al. (2024).

The existing computational models for simulating the processes in the bone-implant interface can be divided into direct and indirect contact models. Direct contact-based models represent bone ingrowth by changing parameter values of the contact elements (Viceconti et al. 2000; Fernandes et al. 2002; Pettersen et al. 2009; Viceconti et al. 2004; Sun et al. 2024). Raffa et al. (2019) suggested a multi-scale approach to account for micromechanical features in the interface. In contrast, indirect contact models consider a separate layer of elements for the interface. For example, spring or interface elements have been used (Tarala et al. 2013; Chanda et al. 2020; Ghosh et al. 2023; Lutz and Nackenhorst 2012; Lutz 2011). Both modelling types offer distinct advantages and disadvantages. Whereas direct models are computationally more efficient, indirect models include more advanced assumptions. However, in reality, the implant is in direct contact with the bone after the implantation (Sun et al. 2024).

Only a few computational models exist to simulate and analyse the femoral stem migration or subsidence (Ovesy and Zysset 2023; Tarala et al. 2013; Pettersen et al. 2009). Pettersen et al. (2009) used a contact algorithm for the bone-stem interface and showed that the implant experiences an initial settling period. Comparably, Tarala et al. (2013) could show the initial subsidence of the implant stem using spring elements for the interface. After this initial settling period the bony ingrowth process is started. A more recent approach has been introduced by Ovesy and Zysset (2023) who use an explicit FEM approach to analyse stem subsidence.

*Scope of the current work*

In this work, a novel approach to simulate the stem migration after total hip replacement is presented. Compared to the few existing approaches, which mostly rely on contact-based formulations, the suggested indirect approach uses the bio-active interface model by Lutz (2011) for the bone-stem interface to analyse the stem migration. Thus, the influence of material parameters in the interface on the migration values can be studied. Lutz (2011) used the interface model only to simulate the osseointegration of the interface layer and plastic motions have not been considered in the previous studies, which in this work are assumed to model the stem migration. The simulations for the migration and osseointegration are decoupled based on the results of Budde et al. (2024), which suggest that the migration ends prior to the onset of the osseointegration. An extensive parameter study is performed and a user material subroutine for Abaqus is provided.

The remainder of the article is structured as follows: in Sect. 2, the material models for migration, osseointegration, and bone remodelling are explained. Next, the numerical implementation of the migration and osseointegration is described in Sect. 3. The results of a sensitivity analysis for the migration and numerical examples for the osseointegration are presented in Sect. 4 and discussed in Sect. 5. Lastly, conclusions are drawn in Sect. 6.

## Bone-stem interface model

A pseudocode for the simulation of the stem migration and osseointegration is depicted in Algorithm 1. Once the model is initialised with an initial bone mass density (BMD) $\varrho$ distribution, the migration of the stem $u_m$ is calculated using a bio-active interface model and a representative force from the gait cycle as well as representative muscle forces. Following this, the interface model is employed to simulate the osseointegration. The stimulus in the interface $\psi _i$ is calculated over representative forces ${\textbf{F}}_i$ from the gait cycle. If the initial micromotions $\tilde{u}_{\text {init}}$ at the interface in the first iteration remain below a predefined threshold $\tilde{u}_{\text {max}}$, the BMD and the degree of osseointegration $\xi$ are updated based on the bone remodelling material model. This process is continued until convergence is reached.

The migration and osseointegration material models are explained in Sects. 2.1 and 2.2. Further, the bone remodelling material model for updating the BMD during the osseointegration process is described in Sect. 2.3.

**Algorithm 1:**
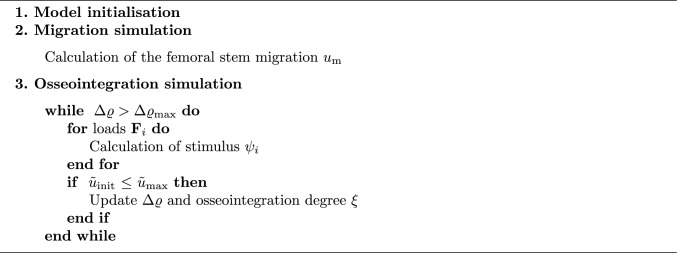
Pseudocode for the simulation of the migration and osseointegration in the bone-stem interface

### Migration model

Following the assumptions from Lutz (2011), the post-operative conditions in the bone-stem interface can be modelled with a Drucker-Prager (DP) plasticity model because initially only compressive forces can be transmitted due to the presence of blood and mushy bone in the interface. Small strain plasticity can be assumed for the interface because only small deformations can occur as the stem is surrounded by bone. Further, large strain plasticity is not used to avoid large element distortion of the interface elements.

The Drucker-Prager yield criterion is formulated as

1 $$\begin{aligned} f_{\text {DP}}&= \Vert \tilde{\varvec{\sigma }} \Vert - \sqrt{2} c - \sqrt{2} \alpha \sigma _m \le 0 \end{aligned}$$

2 $$\begin{aligned}&= \sqrt{J_2} - c - \alpha \sigma _m \le 0 \end{aligned}$$

where $\tilde{\varvec{\sigma }}$ is the deviatoric stress tensor, *c* is a material-dependent adhesion parameter, $\alpha$ is a friction coefficient and $\sigma _m$ is the mean stress. $J_2$ is the second invariant of the deviatoric stress tensor. By setting $\alpha$ to zero, the von Mises yield criterion can be retrieved. The last term incorporates the pressure sensitivity as $\sigma _m = -p$, in analogy to regularised Coulomb friction. Compared to the Coulomb friction law, the model can incorporate interface adhesion. However, the adhesion parameter is set to a small value, allowing only a small tensile load transfer.

A non-associated flow rule, using the von Mises yield criterion as flow potential, is used for the evolution equation for the plastic deviatoric strains

3 $$\begin{aligned} {}^{\text {pl}}{}{\dot{\tilde{\varvec{\varepsilon }}}} = \dot{\lambda } \frac{\partial f}{\partial \tilde{\varvec{\sigma }}} = \dot{\lambda } {\varvec{n}} = \dot{\lambda } \frac{\partial \Vert \tilde{\varvec{\sigma }} \Vert }{\partial \tilde{\varvec{\sigma }}} , \end{aligned}$$

where $\lambda$ is a plastic multiplier and ${\varvec{n}}$ is the flow direction according to the von Mises model (Lutz 2011). $\Vert \cdot \Vert$ denotes the Euclidean norm.

### Osseointegration model

The osseointegration model proposed by Lutz (2011) is briefly explained in the following. For the calculation of the osseointegration, a normalised osseointegration degree depending on the local BMD is introduced as

4 $$\begin{aligned} \xi = \frac{\varrho - \varrho _{\text {min}}}{\varrho _{\text {max}}-\varrho _{\text {min}}} \in [0,1]. \end{aligned}$$

$\varrho _{\text {min}}$ and $\varrho _{\text {max}}$ are the physiological limits of the minimum and maximum BMD. It is assumed that with an increasing osseointegration degree, the Drucker-Prager interface model from Eq. (2) turns into a von Mises interface model. Thus, with an increasing osseointegration degree, representing the bony ingrowth into the stem, more shear and tensile loads can be transmitted. Consequently, the Drucker-Prager von Mises (DPVM) yield function reads

5 $$\begin{aligned} f_{{{\text{DPVM}}}} = & \,\left\| {\tilde{\varvec{\sigma} }} \right\| - \sqrt 2 (c - \alpha \sigma _{{\text{m}}} )(1 - \xi ) \\ & \quad + \sqrt {\frac{2}{3}} \sigma _{{\text{F}}} \xi \le 0, \\ \end{aligned}$$

where $\sigma _F$ is the yield stress.

Directly after the implantation, a high Poisson’s ratio is present due to the mixture of blood and mushy bone in the interface which decreases with the increase in the osseointegration degree. Thus, the Poisson’s ratio is modelled as

6 $$\begin{aligned} \nu = \nu _{\text {max}} - \xi (\nu _{\text {max}} - \nu _{\text {min}}) , \end{aligned}$$

where $\nu _{\text {max}}$ and $\nu _{\text {min}}$ are the maximum and minimum values.

The non-associated flow rule from Eq. (3) is applied here as well.

### Bone remodelling model

The bone remodelling material by Lutz and Nackenhorst (2010) is outlined in the following section. The model is based on the assumptions that the small strain theory is applicable and that the process can be modelled as quasi-static and isothermal.

Further, it is assumed that the free energy density $\psi$ and thus the strain energy density $\Psi$ depend on two internal variables: the elastic strain $\varvec{\varepsilon }$ and the BMD $\varrho$. This relationship is expressed as

7 $$\begin{aligned} \Psi ( \varrho , \varvec{\varepsilon } ) = \varrho \psi ( \varrho , \varvec{\varepsilon } ) . \end{aligned}$$

Following the Clausius-Duhem inequality (Nackenhorst 2018), the Cauchy stress $\varvec{\sigma }$ is derived from the strain energy density

8 $$\begin{aligned} \varvec{ \sigma } = \frac{ \partial \Psi }{ \partial \varvec{ \varepsilon } } . \end{aligned}$$

The non-linear constitutive relation between the BMD and Young’s modulus *E* according to Lutz and Nackenhorst (2010) is used, defined by

9 $$\begin{aligned} E = E_0 \left( \frac{ \varrho }{ \varrho _0 } \right) ^2 , \end{aligned}$$

where $E_0$ and $\varrho _0$ are reference values. This relation is inserted into the generalised Hooke’s law

10 $$\begin{aligned} \varvec{ \sigma } = {\mathbb {C}} :\varvec{ \varepsilon } , \end{aligned}$$

with the linear elastic material tensor ${}^{\text {el}}{{\mathbb {C}}}$ derived for a reference state $\varrho _0$, which leads to the constitutive relation

11 $$\begin{aligned} {\varvec{\sigma }} = \left( \frac{ \varrho }{{\varrho _{0}}} \right) ^{2} {\phantom {0}}^{\text {el}}{{\mathbb {C}}} :{\varvec{\varepsilon }} . \end{aligned}$$

From this relation, the mechanical free energy density $\psi$ is concluded

12 $$\begin{aligned} \psi&= \left( \frac{ \varrho }{ \varrho _0} \right) ^2 \psi _{\text {el}} \nonumber \\&= \frac{1}{\varrho } \left( \frac{ \varrho }{ \varrho _0} \right) ^2 \left[ \frac{\tilde{\lambda }}{2} \text {tr}( \varvec{\varepsilon })^2 + \mu \text {tr} (\varvec{\varepsilon }^2) \right] , \end{aligned}$$

where $\tilde{\lambda }$ and $\mu$ are the Lamé parameters and $\psi _{\text {el}}$ is the linear elastic reference free energy, respectively.

The evolution equation for the BMD, obtained from the balance of mass, is defined as

13 $$\begin{aligned} \dot{\rho } = \frac{\partial \varrho }{\partial t} , \end{aligned}$$

where *t* denotes the process time of the quasi-static simulation. The mass source $\dot{\rho }$ is defined according to the strain energy density driven bone remodelling formulation by Beaupré et al. (1990) using a first-order approach

14 $$\begin{aligned} \dot{\rho } = k \left( \frac{ \Psi }{ \Psi _\text {ref} } -1 \right) . \end{aligned}$$

Here, *k* is a model parameter that describes the speed of the remodelling process and $\Psi _\text {ref}$ is a physiological target value.

## Numerical implementation

For the evolution of Eq. (3), the radial return mapping algorithm is used and will be briefly explained in the following (Lutz and Nackenhorst 2012; Lutz 2011).

First, the non-associated flow rule is discretised in time

15 $${}_{{n + 1}}^{{{\text{pl}}}} \tilde{\varvec{\varepsilon } } = {}_{n}^{{{\text{pl}}}} \tilde{\varvec{\varepsilon } } + \Delta \lambda _{{n + 1}} \varvec{n}{\text{ }}$$

where $\Delta \lambda = {}_{n+1}{\lambda } - {}_{n}{\lambda }$. By inserting this relation, the deviatoric stresses are calculated as

16 $$\begin{aligned} {}_{n+1}{\tilde{\varvec{\sigma }}}&= 2 \mu\; {{}^{\text {el}}_{n+1}}{\tilde{\varvec{\varepsilon }}} \nonumber \\&= 2 \mu ( {}_{n+1}{\tilde{\varvec{\varepsilon }}} - {{}^{\text {pl}}_{n+1}}{\tilde{\varvec{\varepsilon }}}) \nonumber \\&= 2 \mu ( {}_{n+1}{\tilde{\varvec{\varepsilon }}} - {{}^{\text {pl}}_{n}}{\tilde{\varvec{\varepsilon }}}) - 2 \mu \Delta \lambda {}_{n+1}{{\varvec{n}}} \end{aligned}$$

For the computation, a predictor-corrector method is applied, where in a first step, a trial stress known as an elastic predictor is calculated

17 $$\begin{aligned} {{}^{\text {trial}}_{n+1}}{\tilde{\varvec{\sigma }}} = 2 \mu ( {}_{n+1}{\tilde{\varvec{\varepsilon }}} - {{}^{\text {pl}}_{n}}{\tilde{\varvec{\varepsilon }}}) \end{aligned}$$

and consequently the flow rule in Eq. (5) is evaluated

18 $$\begin{aligned} _{{n + 1}}^{{{\text{trial}}}} f = & \Vert {_{{n + 1}}^{{{\text{trial}}}} \tilde{\varvec{\sigma} }} \Vert - \sqrt 2 (c - \alpha \sigma _{m} )(1 - \xi ) \\ & \quad + \sqrt {\frac{2}{3}} \sigma _{F} \xi \le 0. \\ \end{aligned}$$

If ${{}^{\text {trial}}_{n+1}}{f} \le 0$ the assumption of the elastic predictor is correct. However, if ${{}^{\text {trial}}_{n+1}}{f} > 0$, the stress needs to be projected onto the yield surface

19 $$\begin{aligned} {}_{n+1}{\tilde{\varvec{\sigma }}} = {{}^{\text {trial}}_{n+1}}{\tilde{\varvec{\sigma }}} - 2 \mu \Delta \lambda {}_{n+1}{{\varvec{n}}} \end{aligned}$$

where ${}_{n+1}{{\varvec{n}}} = {{}^{\text {trial}}_{n+1}}{{\varvec{n}}}$ because ${{}^{\text {trial}}_{n+1}}{\tilde{\varvec{\sigma }}}$ and ${}_{n+1}{\tilde{\varvec{\sigma }}}$ are collinear. The plastic multiplier $\Delta \lambda$ is calculated by rewriting the equation as

20 $$\begin{aligned} \Vert {}_{n+1}{\tilde{\varvec{\sigma }}} \Vert = \Vert {{}^{\text {trial}}_{n+1}}{\tilde{\varvec{\sigma }}} \Vert - 2 \mu \Delta \lambda \end{aligned}$$

which is inserted in the yield function $_{n+1}{f}$

21 $$\begin{aligned} {}_{n+1}{f} = {{}^{\text {trial}}_{n+1}}{f} - 2 \mu \Delta \lambda = 0 \end{aligned}$$

and results in

22 $$ \Delta \lambda  = \frac{{{}_{{n + 1}}^{{{\text{trial}}}} f}}{{2\mu }}.{\text{ }} $$

The algorithmic consistent tangent operator is defined as

23 $$\begin{aligned} {}^{\text {ep}}{{\mathbb {C}}}&= {{}^{\text {el}}_{\text {vol}}}{{\mathbb {C}}} + {}^{\text {ep}}{\tilde{{\mathbb {C}}}} :{\mathbb {P}} \end{aligned}$$

24 $$\begin{aligned} & \, = \kappa \varvec{I} \otimes \varvec{I} \\ & \quad + 2\mu \left( {\underbrace {{\left[ {1 - \Delta \lambda \frac{{2\mu }}{\Vert {{}^{\text {trial}}_{n+1}}{\tilde{\varvec{\sigma }}} \Vert }} \right]\left[ {{\mathbb{P}} - _{{n + 1}}^{\text{trial}}\varvec{n} \otimes _{{n + 1}}^{{{\text{trial}}}}\varvec{n}} \right]}}_{{_{{vM}}^{{{\text{ep}}}} \tilde{\mathbb{C}}}}} \right. \\ & \left. \quad - (1 - \xi )\hat{\varvec{n}} \otimes _{{n + 1}}^{{{\text{trial}}}}\varvec{n}) \right) \\ \end{aligned}$$

where the deviatoric projection tensor

25 $$\begin{aligned} {\mathbb {P}} = {\mathbb {I}} - \frac{1}{3} {\varvec{I}} \otimes {\varvec{I}} \end{aligned}$$

and

26 $$\begin{aligned} \hat{{\varvec{n}}} = \frac{1}{3\sqrt{2}\mu } \alpha {\varvec{I}} :{}^{\text {el}}{}{{\mathbb {C}}} = \text {const.} \end{aligned}$$

${{}^{\text {ep}}_{\text {vM}}}{\tilde{{\mathbb {C}}}}$ is the tangent of the von Mises plasticity model.

To retrieve the Drucker–Prager model used for the migration, $\xi$ is set to zero. For $\xi = 0$ and if the trial stress is in the complementary cone, the stress has to be projected onto the apex, where ${}_{n+1}{\tilde{\varvec{\sigma }}} = 0$. This relation is inserted into the yield function and thus results in

27 $$\begin{aligned} {{}^{\text {apex}}_{n+1}}{\varvec{\sigma }} = \frac{c (1-\xi ) + \frac{\sigma _f}{\sqrt{3}} \xi }{\alpha (1-\xi ) } {\varvec{1}} \end{aligned}$$

and with $\xi = 0$ leads to

28 $$\begin{aligned} {{}^{\text {apex}}_{n+1}}{\varvec{\sigma }} = \frac{c }{\alpha } {\varvec{1}} . \end{aligned}$$

Because of the vanishing deviatoric stress the plastic multiplier can be calculated as

29 $$\begin{aligned} \Delta \lambda = \frac{\Vert {{}^{\text {trial}}_{n+1}}{\tilde{\varvec{\sigma }}} \Vert }{2 \mu} \end{aligned}$$

which by substituting into Eq. (15) leads to

30 $$\begin{aligned} {{}^{\text {pl}}_{n+1}}{\tilde{\varvec{\varepsilon }}}&= {{}^{\text {pl}}_{n}}{\tilde{\varvec{\varepsilon }}} + \Delta \lambda \frac{{{}^{\text {trial}}_{n+1}}{\tilde{\varvec{\sigma }}}}{\Vert {{}^{\text {trial}}_{n+1}}{\tilde{\varvec{\sigma }}} \Vert } \end{aligned}$$

31 $$\begin{aligned}&= {{}^{\text {pl}}_{n}}{\tilde{\varvec{\varepsilon }}} + \frac{{{}^{\text {trial}}_{n+1}}{\tilde{\varvec{\sigma }}}}{2 \mu } \end{aligned}$$

32 $$\begin{aligned}&= {{}^{\text {pl}}_{n}}{\tilde{\varvec{\varepsilon }}} + {}_{n+1}{\tilde{\varvec{\varepsilon }}} - {{}^{\text {pl}}_{n}}{\tilde{\varvec{\varepsilon }}} . \end{aligned}$$

Further, the algorithmic consistent tangent operator vanishes

33 $$\begin{aligned} {{}^{\text {apex}}_{n+1}}{{\mathbb {C}}} = 0 . \end{aligned}$$

For a detailed explanation and derivation of the numerical treatment, it is referred to Lutz and Nackenhorst (2012) and Lutz (2011).

## Numerical results

First, the finite element model is explained in Sect. 4.1. The results of the migration simulation are presented in Sect. 4.2, followed by a numerical example illustrating the influence of the migration on the osseointegration in Sect. 4.3.

### Finite element model

**Fig. 1:**
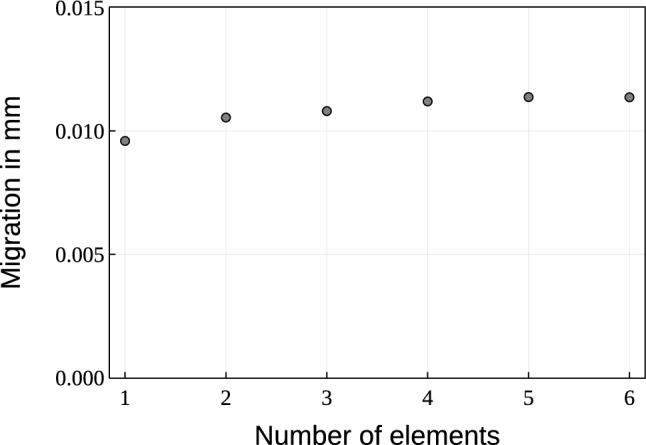
Migration $u_m$ in mm for reference parameters ($\alpha =0.6, c=0.5\,{\text{N}}/{\text{mm}^2}, k_F=1.0, d=1.0\,\text {mm}, \varrho _{\text {init}}=0.048\, {\text{g}}/{\text {cm}^3}$) for different number of elements in the interface

The geometry of the human femur with an integrated non-cemented Metha^®^ stem (Aesculap, Tuttlingen, Germany), as described in Lutz (2011) and Bensel et al. (2023), is used. Further, a thin interface layer surrounding the implant stem is added. The migration values have been analysed for the reference parameters for different numbers of interface elements in Fig. 1. To avoid numerical instabilities, one element layer is chosen as a suitable engineering approach. The stem is divided into a structured proximal part, where osseointegration is possible, and a smooth (polished) distal part.

**Fig. 2:**
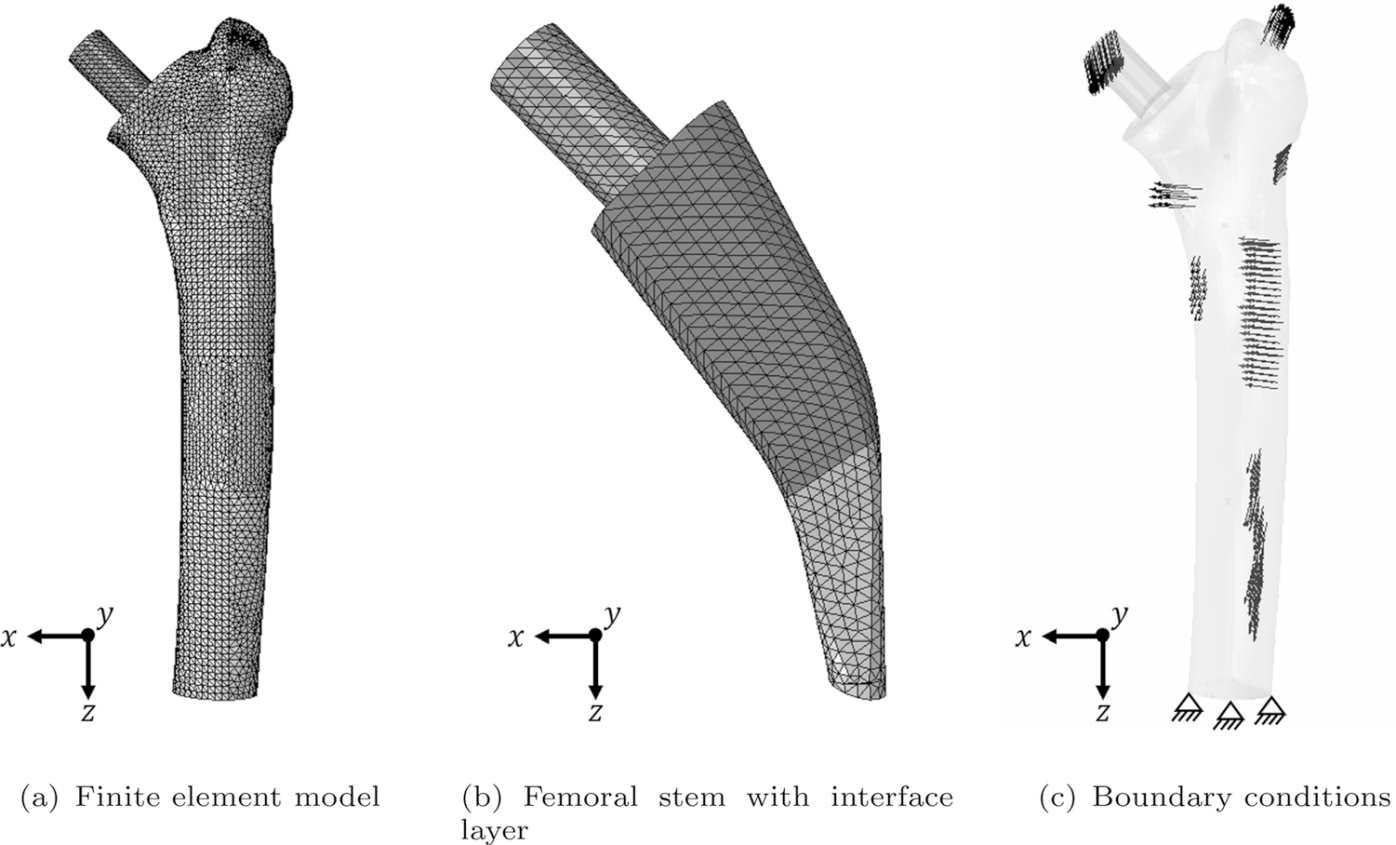
FEM model with **a** FEM mesh **b** FEM mesh of the stem with interface elements (proximal active and distal inactive interface elements) **c** boundary conditions (joint load and muscle forces)

To accurately represent the geometry, a fine mesh size similar to that used by Bensel et al. (2023) is uniformly applied. Both the stem and the surrounding bone are meshed with linear tetrahedral elements, while the interface is modelled with a single layer of linear wedge interface elements. For the subsequent parameter study, the thickness of the interface *d* is varied. The number of elements for the different meshes is depicted in Table 1. Notably, the number of elements for the stem and the interface layer remain constant for all meshes. Exemplary, the resulting FEM mesh for $d = 0.75$ mm is depicted in Fig. 2a, with a close-up view on the stem and the interface in Fig. 2b. In this detailed view, the active proximal and the inactive distal part are highlighted.

**Table 1:** Number of elements for FEM models with different interface thickness *d* in mm (total number of elements $N_{\text {all}}$, number of tetrahedral elements $N_{\text {tet}}$, number of wedge elements $N_{\text {wed}}$)

*d*	$N_{\text {all}}$	$N_{\text {tet}}$	$N_{\text {wed}}$
0.1	108143	106306	1837
0.5	105288	103451	1837
0.75	103994	102157	1837
1.0	102469	100632	1837

The initial and boundary conditions are adopted from Lutz (2011). To establish the initial BMD distribution of the femur, the biomechanically equilibrated BMD distribution for the complete femur (including the femur head) is projected onto the model with the femoral stem. The boundary conditions represent a clamping at the bottom and loading by the joint force as well as the six main muscle forces (see Fig. 2c). The muscle loads have been calculated as statically equivalent loads corresponding to the measured BMD distribution projected from CT data to the FEM model (Lutz 2011). The values for the muscle forces are depicted in Table 2. The muscle forces play a minor role as the joint force is directly acting on the stem (Lutz and Nackenhorst 2012).

For the numerical solution, the commercial FEM software Abaqus (Abaqus 2017, Dassault Systèmes, Vélizy-Villacoublay, France) is used. The Young’s modulus of the femur elements is calculated according to Eq. (9) with $$E_0 = 6500 $$ $$ \text {N/mm}^2 $$ and a Poisson’s ratio $$\nu = 0.31$$ using a linear elastic user material (UMAT) subroutine. For the stem, a linear elastic material of titanium with a Young’s modulus $$E = 105000 $$ $$ \text {N/mm}^2 $$ and a Poisson’s ratio $$\nu = 0.31$$ are used. The bio-active interface model has been implemented as a UMAT and the parameters used are mentioned in the following sections.

**Table 2:** Static equivalent muscle forces in N from Lutz (2011)

Force	${F}_{x}$	${F}_{y}$	${F}_{z}$	${F}_{R}$
Gluteus medius / minimus	$-$89.2	103.7	$-$222.4	261.1
Vastus lateralis	146.9	439.8	96.8	473.7
Iliopsoas	193.0	7.7	$-$16.4	193.8
Biceps femoris	18.7	$-$93.4	54.4	109.7
Gluteus maximus	266.2	143.4	$-$34.2	304.3
Vastus medialis	39.3	$-$397.0	150.8	426.5

### Migration

For the simulation of the migration of the hip implant stem, a joint force of ${\textbf{F}}$ = [−533.2 −344 2369] N is applied which corresponds to the largest resultant force in the gait cycle. In a first step, the load is applied, and in the second step, the load is removed to determine the stem migration $u_{m}$ resulting from the plastic deformations.

In accordance with Budde et al. (2024), the migration $u_m$ is calculated at the geometric centre of the femoral stem and is defined as

34 $$\begin{aligned} u_\text {m} = \sqrt{ {{}^{\text {pl}}{}{u}_x}^2 + {{}^{\text {pl}}{}{u}_y}^2 + {{}^{\text {pl}}{}{u}_z}^2 } , \end{aligned}$$

where ${{}^{\text {pl}}{}{u}_i}$ are the plastic deformations in x-, y-, and z-direction.

Several migration simulations with different parameter combinations are performed for the global sensitivity analysis. For this analysis, the adhesion coefficient *c*, the friction coefficient $\alpha$ of the active interface, the force factor $k_F$, which is multiplied by the predefined joint load, the initial BMD in the interface $\varrho _{\text {init}}$, which corresponds to the initial Young’s modulus of the interface layer (interface stiffness) as well as the interface thickness *d* are varied. The parameters for the parameter study are depicted in Table 3 and are varied around the parameters defined by Lutz (2011). For $\varrho _{\text {init}}$, the maximum, centre, and minimum value according to the parameter study by Lutz (2011) are chosen. Consequently, 324 migration simulations are performed and the total migration at the geometric centre of the implant stem is calculated.

**Table 3:** Material parameters for the migration simulation

Parameter	Value	Unit
$\alpha _\text {active}$	$\in \{0.25, 0.6, 1.0\}$	[-]
$\alpha _\text {inactive}$	0.25	[-]
$c_\text {active}$	$\in \{0.25, 0.5, 1.0 \}$	[$\text {N}/\text {mm}^2$]
$c_\text {inactive}$	0.25	[$\text {N}/\text {mm}^2$]
$k_F$	$\in$ {0.5, 1.0, 2.0}	[-]
$\varrho _\text {init}$	$\in \{0.039, 0.048, 0.062\}$	[$\text {g}/\text {cm}^3$]
*d*	$\in \{0.1, 0.5, 0.75, 1.0\}$	[$\text {mm}$]
$\nu _\text {min}$	0.29	[-]
$\nu _\text {max}$	0.45	[-]

**Fig. 3:**
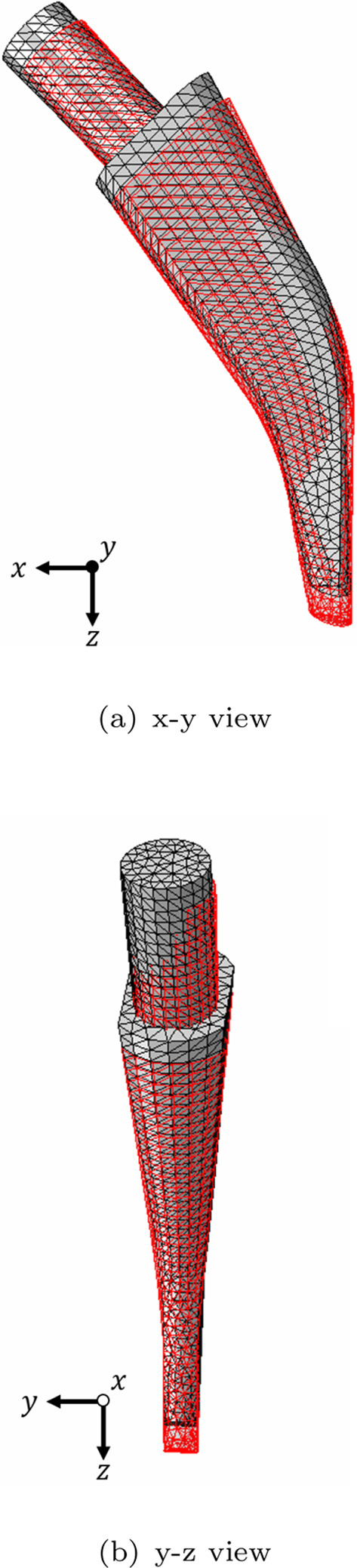
x-y view **a** and y-z view **b** of original stem position (grey) and deformed stem position (red). The deformation scale factor has been set to 20

The maximum migration for the tested parameter combinations is 0.27 mm for the parameter combination $\alpha =0.25, c=0.25\,{\text{N}}/{\text{mm}^2}, k_F=2.0, d=1.0~\text {mm}, \varrho _{\text {init}}=0.039\, {\text{g}}/{\text {cm}^3}$. In Fig. 3, the original and the migrated stem position considering the permanent displacements are shown. It can be seen that the stem moves downwards and performs a rotational movement.

**Fig. 4:**
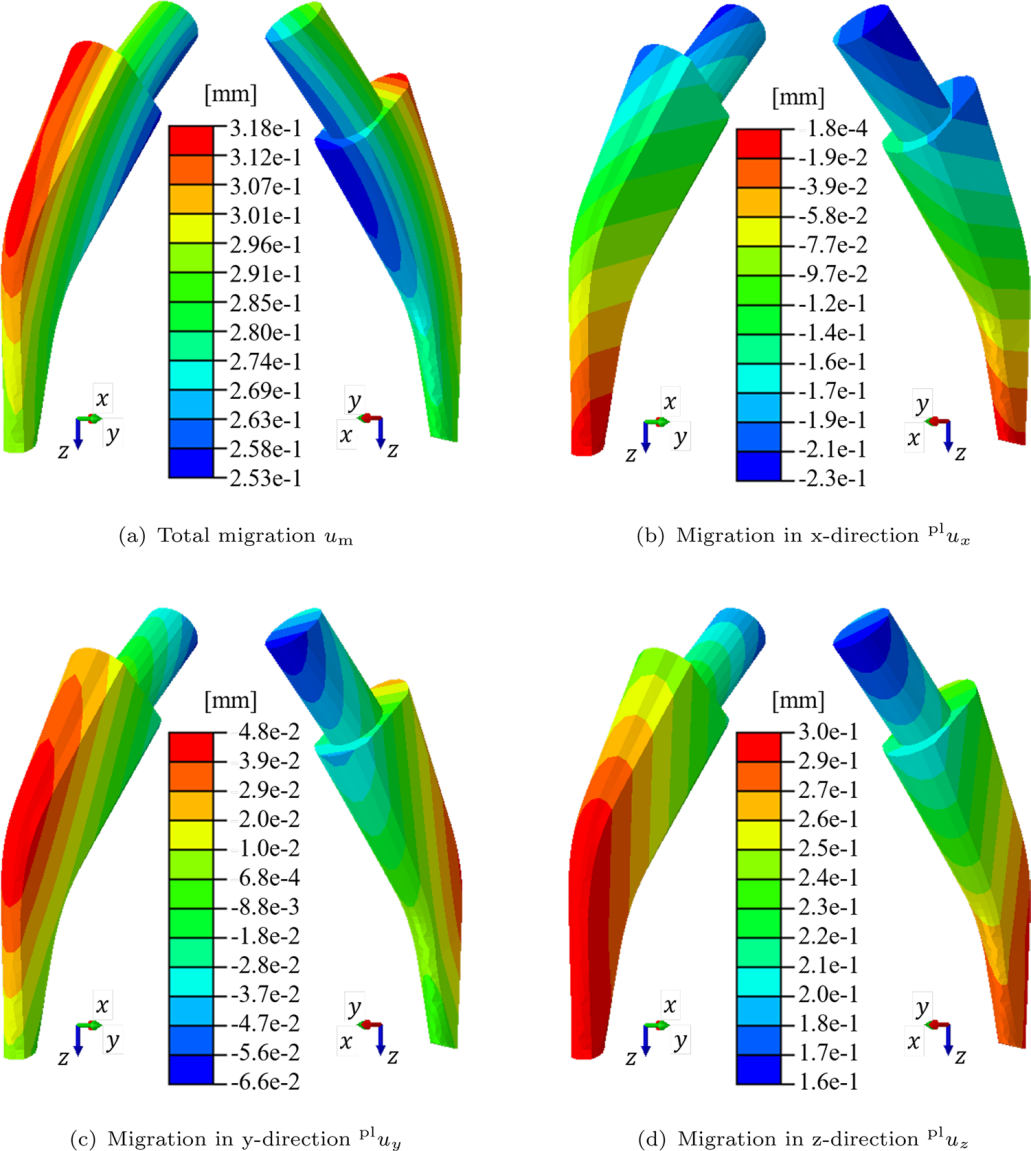
Migration of the implant stem in mm. **a** total migration and migration **b** in x-direction, **c** in y-direction and **d** in z-direction

**Fig. 5:**
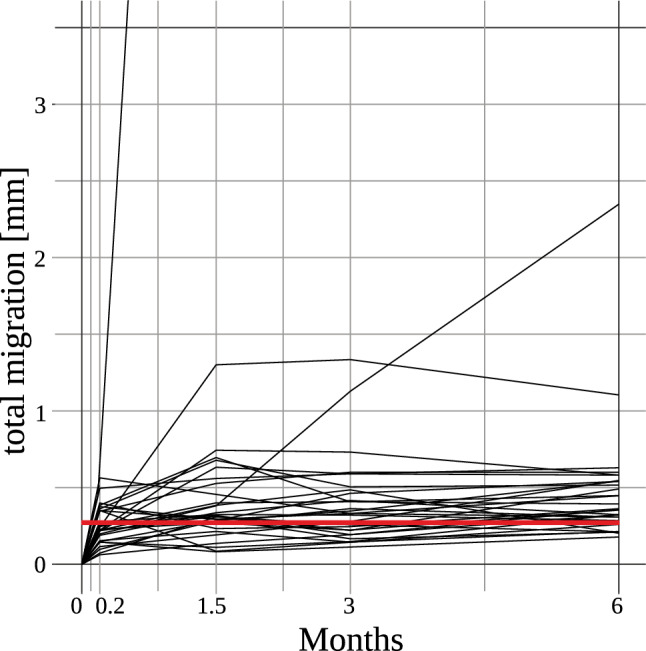
Total migration for the clinical and numerical study. Black: migration in the first six months after baseline for all patients from A2 stem with titanium plasma spray coating from clinical study of Budde et al. (2024). Red: maximum migration from the numerical simulation with the Metha stem

The migration values according to Eq. (34) are depicted in Fig. 5. The total migration of the implant stem as well as the permanent displacements in x-, y-, and z-direction are depicted in Fig. 4. The largest total migration in Fig. 4a is present at the lateral face of the stem with around 0.3 mm, whereas the smallest values are present at the medial face. Regarding the contributions to the total migration, the displacements in x- and y-direction show a minor contribution (see Figs. 4b and c). In contrast, the displacement in z-direction (see Fig. 4d) has the largest contribution with high values in the distal part and smaller values in the proximal part of the stem which is due to the rotation of the stem (see Fig. 3). This observation aligns with the frequently mentioned phenomenon of stem subsidence and can be explained by the large joint force in z-direction (Floerkemeier et al. 2020).

In Fig. 5, the maximum simulated migration is sketched with data for the migration of A2 stems obtained by Budde et al. (2024). While different parameter combinations might result in an increased migration, the computed maximum migration is in the same magnitude as the migration values observed in clinical practice. For more details on the clinical study design, it is referred to the aforementioned study.

For the sensitivity analysis, the "Spearman’s rank correlation coefficient", which considers non-linear dependencies based on the rank of the sample output, is defined as

35 $$\begin{aligned} \rho = \frac{\text {cov} (R(Y), R(X)) }{\sigma _{R(X)} \sigma _{R(Y)}} , \end{aligned}$$

where $\text {cov} (R(Y), R(X))$ is the covariance of the rank variables and $\sigma _{R(X)}$ and $\sigma _{R(Y)}$ are the standard deviations of the rank variables.

**Fig. 6:**
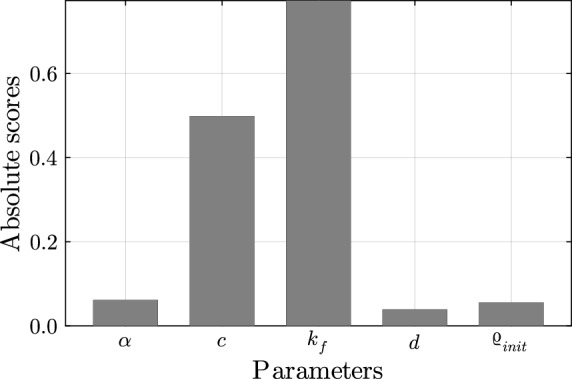
Cross-correlation for parameters of the migration simulation

The results of the sensitivity analysis are depicted in Fig. 6. The change in the loading factor has the largest influence on the migration. When the loading increases, the migration also increases. The adhesion parameter is the second most influential parameter. A lower adhesion coefficient leads to more migration, as plastic flow occurs earlier (see Eq. (2)). The remaining parameters have a lesser impact on the total migration of the implant stem. Specifically, the friction coefficient and the initial BMD at the interface have inverse effects, while the interface thickness has a positive influence. Budde et al. (2024) demonstrated in their study that adding a calcium phosphate coating to the stem resulted in greater migration despite the coating being intended to enhance the bone ingrowth and thereby decrease the migration. The authors of the study proposed several explanations for the decreasing migration, including that the coating reduced friction, which aligns with the observed effects of the friction coefficient. The influence of the initial BMD in the interface is attributed to the initial stiffness of the interface. The thickness can be compared with the initial press-fit as the tighter the press-fit is, the smaller the migration should be.

**Fig. 7:**
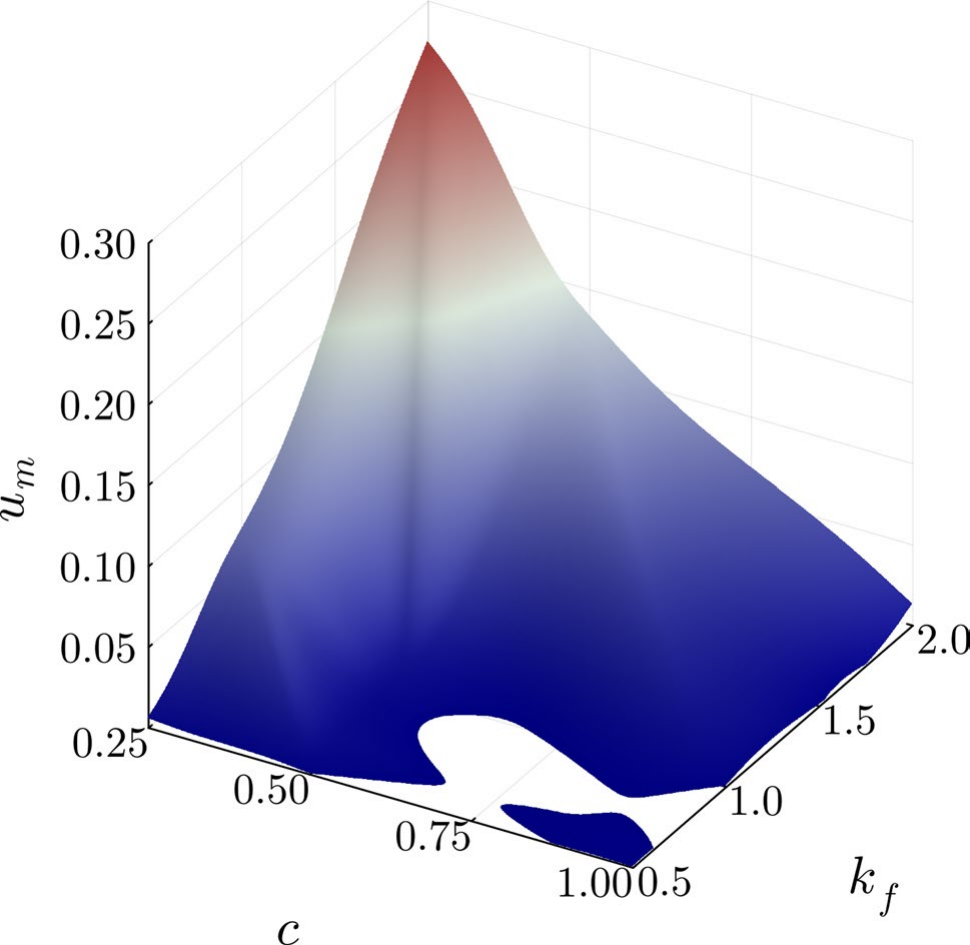
Total migration $u_m$ in mm for the adhesion parameter *c* and the loading factor *k*_*f*_

In Fig. 7, the total migration is plotted for the two most influential parameters, the adhesion factor *c*, and the loading factor $k_f$. The remaining parameters were kept constant with $\alpha _{\text {active}} = 0.25$, $d = 1.0$ mm and $\varrho _{\text {init}}$ = 0.039 $\text {N}/\text {mm}^2$. The depicted surface plot confirms the aforementioned influence of these parameters on the total migration. Further, a non-linear relationship becomes visible.

### Osseointegration

To analyse the influence of the stem migration on the osseointegration process, one simulation with the maximum migrated stem position and one simulation with the original stem position are performed. The material parameters for the osseointegration simulation are shown in Table 4. For a detailed study on the influence of the material parameters on the osseointegration, it is referred to Lutz (2011).

For the osseointegration simulation, two forces, representing the mean stimulus according to Lutz (2011), are successively applied ($F_1 = [-447.2~ -\!74.2 ~1717.8]$ N and $F_2 = [-181.8~ 35.6~ 365.3]$ N) as shown in Algorithm 1.

**Table 4:** Material parameters for the osseointegration simulation

Parameter	Value	Unit
$\alpha _\text {active}$	0.25	[-]
$\alpha _\text {inactive}$	0.25	[-]
$c_\text {active}$	0.25	[$\text {N}/\text {mm}^2$]
$c_\text {inactive}$	0.25	[$\text {N}/\text {mm}^2$]
$\varrho _\text {init}$	0.039	[$\text {g}/\text {cm}^3$]
*d*	1.0	[$\text {mm}$]
$\nu _\text {min}$	0.29	[-]
$\nu _\text {max}$	0.45	[-]
$\sigma _F$	20	[$\text {N}/\text {mm}^2$]
$\varrho _\text {min}$	0.039	[$\text {g}/\text {cm}^3$]
$\varrho _\text {max}$	2.0	[$\text {g}/\text {cm}^3$]
$\Psi _\text {ref}$	0.0003	[$\text {N}/\text {mm}^2$]
*k*	0.3	[-]
$\tilde{u}_\text {max}$	100	[$\mu$m]

The micromotions in the interface are computed by considering the in-plane micromotions for the loaded and unloaded configuration and taking the average for both forces. The micromotions are only analysed in the active part of the interface and are an indicator of whether osseointegration is possible.

**Fig. 8:**
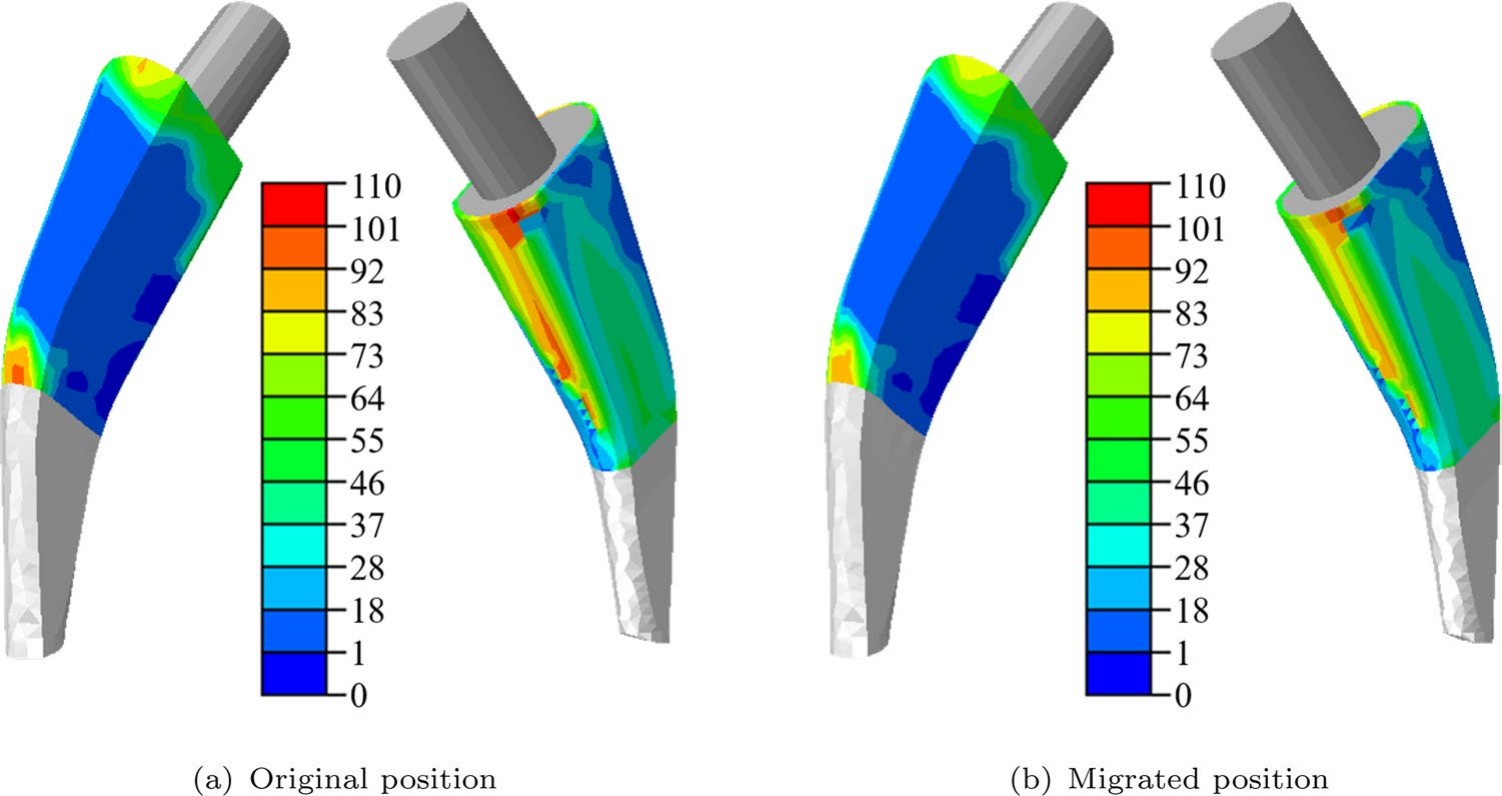
Micromotions in $\mu$m in the active bone-stem interface for original **a** and migrated **b** stem position

The resulting micromotions are depicted in Fig. 8. In general, the micromotions depict a similar pattern for both positions. The largest micromotions are present at the lateral side of the stem and at the proximal medial and distal lateral sides. Further, higher micromotions are found at the anterior side of the stem. For the maximum values, a value of 100 $\mu$m for the migrated and a value of 106 $\mu$m for the original position are found. As a result, it can be concluded that the micromotions decrease with the increasing migration of the implant stem. This decrease in micromotions could lead to more osseointegration because no osseointegration is possible if the micromotions exceed a certain limit.

**Fig. 9:**
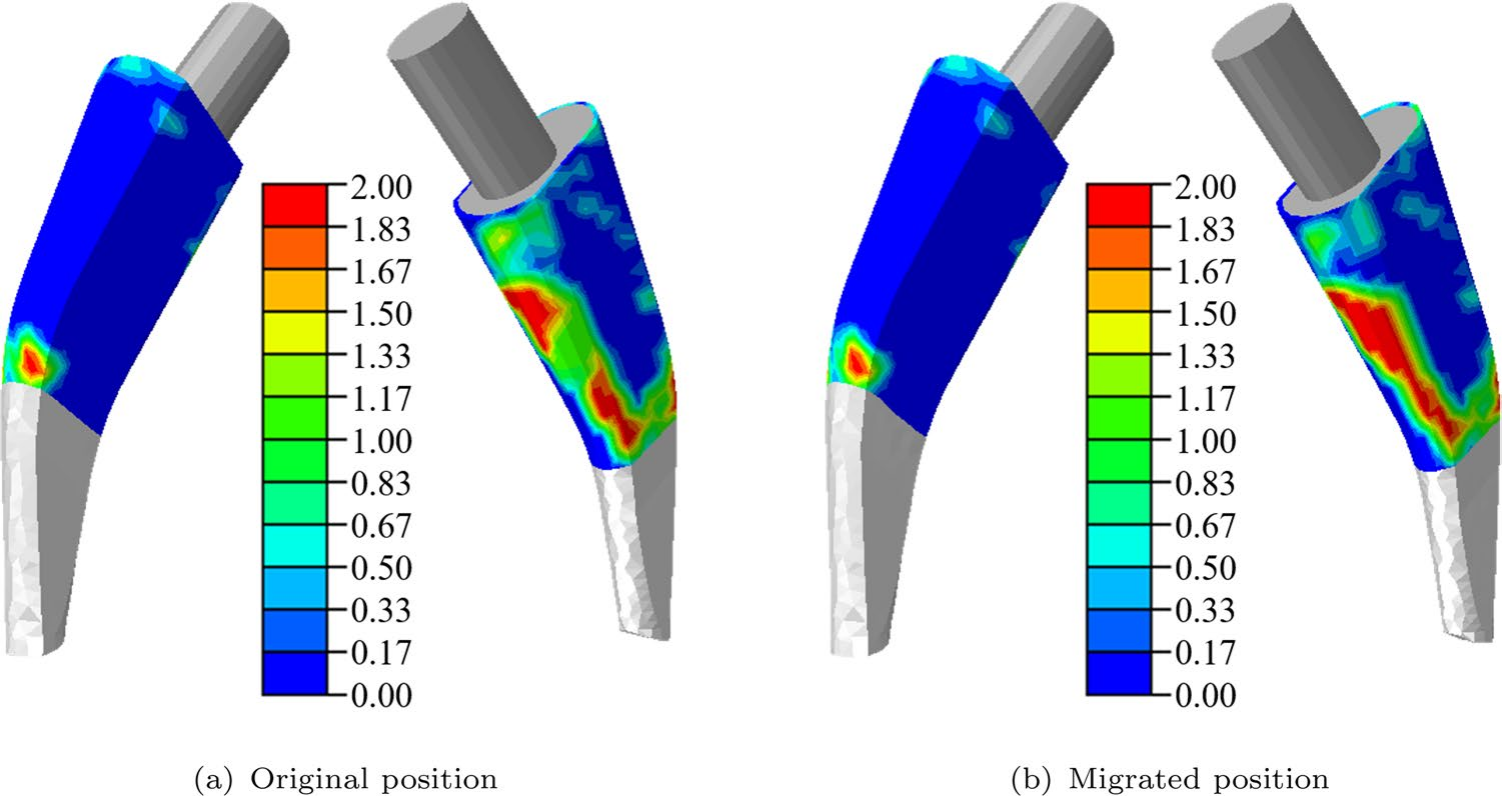
BMD distribution in $\text {g}/\text {cm}^3$ after the osseointegration in active bone-stem interface for original **a** and migrated **b** stem position

In Fig. 9, the BMD distribution after osseointegration in the active bone-stem interface is depicted for the original and migrated position. The micromotion limit has been set to 100 $\mu$m. Similar osseointegration patterns are present with most of the osseointegration occurring at the lateral side of the stem. The osseointegration distribution is similar to the distribution of the micromotions (see Fig. 8), suggesting that micromotions below the limit promote osseointegration whereas no micromotions lead also to no osseointegration. The osseointegration is less pronounced for the original position which can be attributed to the micromotion limit.

## Discussion

Using the bio-active interface model for the bone-stem interface, it is possible to depict the stem migration after total hip replacement. The computational model uses the bio-active interface model by Lutz (2011), who proposed a mixed Drucker–Prager von Mises plasticity model for the interface to model the osseointegration, where the interface model was only used to simulate the osseointegration of the interface layer and plastic motions have not been considered, which in this work are assumed to depict the stem migration. Compared to the existing approaches to model stem migration, parameter studies on the different parameters in the constitutive model can be performed. Further, the model has been implemented as an open-access UMAT subroutine in Abaqus.

The migration and osseointegration simulations have been decoupled based on the study by Budde et al. (2024). The study indicated that early migration ends before the completion of osseointegration, and therefore, these processes can be regarded separately. It was observed that 65% of the femoral stems reached their final position and ceased migrating after just one week. Furthermore, by six weeks, 87% reached their final positions. Only a few stems experienced a more substantial migration afterwards, which justifies the decoupling. The same decoupling approach has also been followed by Tarala et al. (2013).

The calculation of the migration is often defined differently. In this study, the migration was calculated using the magnitude of the permanent displacement in the geometric centre of the stem. Other studies only consider, e.g., the subsidence of the stem which is the permanent displacement in the vertical direction. In the presented results, the subsidence also has the largest contribution to the total migration (see Fig. 4d).

The computed migration values are in the same magnitude as those presented in clinical and numerical studies (Budde et al. 2024; Tarala et al. 2013; Pettersen et al. 2009; Ovesy and Zysset 2023). The numerical studies reported migration values up to 0.4 mm which aligns well with our study. After six months, exemplary stems showed the following average subsidence values: Metha stem 0.83 mm, A2 stem 0.33 mm, or Optimys stem 0.21 mm (Budde et al. 2024). Present differences can be attributed to different initial and boundary conditions. Further, most of the stems experience a small migration and only a few stems contribute to the larger average migration (Budde et al. 2024).

As only limited knowledge is available on the bone-implant interface, i.e., the material parameters of the interface, assumptions are required for the computational model. For example, a specific micromotion limit for the osseointegration still needs to be determined (Sun et al. 2024). Moreover, an indirect model using an interface layer has been used for the bone-stem interface. However, Sun et al. (2024) states that, in reality, the implant is in direct contact with the surrounding bone. In the current study, several interface thicknesses have been analysed and the influence on the migration has been found to be minor (see Fig. 6).

The advantage of the current indirect model compared to the existing approaches is that by using an interface layer a constitutive model can be chosen for the interface. Thus, the computational model offers the possibility of analysing the influence of different parameters, such as the influence of different coatings. A sensitivity analysis was conducted on different parameter combinations, revealing that the joint load and the adhesion parameter have the highest influence on the total migration. Due to the elastoplasticity model, the highest load is responsible for the maximum migration. In future analysis, a randomised input could be used for the parameter study. In addition to the elasto-plasticity of the interface, other factors, such as time-dependency, may influence the stem migration and could be included in future modelling approaches.

A brief analysis was conducted on the influence of the migration on the micromotions in the interface which influence the subsequent osseointegration. The numerical example showed that the micromotions and the osseointegration decreased for the migrated position compared to the original position but only minor differences are present. The result shows that a limited migration poses no difficulty for hip implants as a small migration is present for all femoral stems.

The interest in patient-specific models is continuously growing and computational simulations of individual stem migration could be used to guide decision-making in clinical practice. Before the usage in the clinical practice, *in silico* models require a proper validation. However, no RSA data with the corresponding image data were available for the Metha implant to compare the simulation results with clinical data. Furthermore, the validation of the osseointegration model remains a challenge because of the lack of clinical and experimental data (Sun et al. 2024).

### Limitations

The presented model can be used to model small stem migration for hip implants. However, this study still bears some limitations:

- Despite the qualitative agreement with clinical data, the model lacks quantitive validation. For the model to have meaningful clinical impact, thorough validation against experimental or patient-specific data is essential.
- This study employs a generic stem and femur geometry. However, anatomical variability, the initial BMD distribution, and implant positioning as well as the amount of press-fit can also influence the migration behaviour. Future work could incorporate patient-specific geometries and loading conditions and could be combined with a thorough validation.
- The current interface model assumes small deformations, which limits its applicability to low migration scenarios. In clinical practice, high migration values, e.g., up to 1 cm, occasionally occur for individual implants (Budde et al. 2024). To model large elasto-plastic deformations, other simulation methods are necessary (Bonet and Wood 1997). Implementing these approaches, while computationally more demanding, would enable the application of the model in scenarios with high migration values.

## Conclusions

In conclusion, a new approach to simulate the migration of femoral stems after total hip replacement has been presented. Using a bio-active interface model, the computational model delivers migration predictions that align with clinical and numerical studies. The maximum predicted total migration is up to 0.27 mm within the examined parameter ranges. The indirect model bears the advantage to analyse the effect of interface parameters on the migration compared to existing approaches. Further, the model has been implemented as an open-access UMAT subroutine in Abaqus. However, the applicability of the current model is limited to small deformations, indicating the need for alternative methods to depict larger deformations. Nonetheless, the computational model can be used to gain insights into the processes responsible for the stem migration and osseointegration which could potentially contribute to reducing the stem migration in the clinical practice.

## Data Availability

An exemplary UMAT Abaqus subroutine is available under the aforementioned link.

## References

[CR1] Beaupré GS, Orr TE, Carter DR (1990) An approach for time-dependent bone modeling and remodeling-theoretical development. J Orthop Res 8(5):651–661. 10.1002/jor.1100080506 2388105 10.1002/jor.1100080506

[CR2] Bensel F, Reiber M, Foulatier E, Junker P, Nackenhorst U (2023) A gradient-enhanced bone remodelling approach to avoid the checkerboard phenomenon. Comput Mech 73(6):1335–1349. 10.1007/s00466-023-02413-9

[CR3] Bonet J, Wood RD (1997) Nonlinear continuum mechanics for finite element analysis. Cambridge University Press

[CR4] Budde S, Derksen A, Hurschler C, Fennema P, Windhagen H, Plagge J, Flörkemeier T, von Lewinski G, Noll Y, Schwarze M (2024) Very early migration of a calcar-guided short stem: a randomized study of early mobilization and the influence of a calcium phosphate coating with 60 patients. Sci Rep 14(1):3837. 10.1038/s41598-023-50829-3 38360840 10.1038/s41598-023-50829-3 PMC10869691

[CR5] Budde S, Seehaus F, Schwarze M, Hurschler C, Floerkemeier T, Windhagen H, Noll Y, Ettinger M, Thorey F (2016) Analysis of migration of the nanos^®^ short-stem hip implant within two years after surgery. Int Orthop 40:1607–1614. 10.1007/s00264-015-2999-9 26404094 10.1007/s00264-015-2999-9

[CR6] Carter DR, Orr TE, Fyhrie DP (1989) Relationships between loading history and femoral cancellous bone architecture. J Biomech 22(3):231–244. 10.1016/0021-9290(89)90091-2 2722894 10.1016/0021-9290(89)90091-2

[CR7] Chanda S, Mukherjee K, Gupta S, Pratihar DK (2020) A comparative assessment of two designs of hip stem using rule-based simulation of combined osseointegration and remodelling. Proc Inst Mech Eng [H] 234(1):118–128. 10.1177/0954411919890998 10.1177/0954411919890998 31774362

[CR8] Dittrich H, Schimmack M, Siemsen CH (2019) Orthopädische Biomechanik: Einführung in die Endoprothetik der Gelenke der unteren Extremitäten, 1st edn. Springer Vieweg Berlin, Heidelberg

[CR9] Doblaré M, García J (2002) Anisotropic bone remodelling model based on a continuum damage-repair theory. J Biomech 35(1):1–17. 10.1016/S0021-9290(01)00178-6 11747878 10.1016/s0021-9290(01)00178-6

[CR10] Ferguson RJ, Palmer AJ, Taylor A, Porter ML, Malchau H, Glyn-Jones S (2018) Hip replacement. The Lancet 392(10158):1662–1671. 10.1016/S0140-6736(18)31777-X 10.1016/S0140-6736(18)31777-X 30496081

[CR11] Fernandes P, Folgado J, Jacobs C, Pellegrini V (2002) A contact model with ingrowth control for bone remodelling around cementless stems. J Biomech 35(2):167–176. 10.1016/S0021-9290(01)00204-4 11784535 10.1016/s0021-9290(01)00204-4

[CR12] Floerkemeier T (2021) Patientenspezifische Planung in der Hüftendoprothetik. Arthroskopie 34:377–384. 10.1007/s00142-021-00461-y

[CR13] Floerkemeier T, Budde S, Gv L, Windhagen H, Hurschler C, Schwarze M (2020) Greater early migration of a short-stem total hip arthroplasty is not associated with an increased risk of osseointegration failure: 5th-year results from a prospective RSA study with 39 patients, a follow-up study. Acta Orthop 91(3):266–271. 10.1080/17453674.2020.1732749 32106733 10.1080/17453674.2020.1732749 PMC8023937

[CR14] Floerkemeier T, Schwarze M, Hurschler C, Gronewold J, Windhagen H, von Lewinski G, Budde S (2017) The influence of tribological pairings and other factors on migration patterns of short stems in total hip arthroplasty. Biomed Res Int 2017(1):8756432. 10.1155/2017/8756432 28497067 10.1155/2017/8756432 PMC5406728

[CR15] Gao X, Fraulob M, Haïat G (2019) Biomechanical behaviours of the bone-implant interface: a review. J R Soc Interface 16(156):20190259. 10.1098/rsif.2019.0259 31362615 10.1098/rsif.2019.0259 PMC6685012

[CR16] Ghosh R, Hazra A, Chanda S, Chakraborty D (2023) Computational assessment of growth of connective tissues around textured hip stem subjected to daily activities after THA. Med Biol Eng Comput 61(2):525–540. 10.1007/s11517-022-02729-3 36534373 10.1007/s11517-022-02729-3

[CR17] Huiskes R, Weinans H , van Rietbergen B (1992) The relationship between stress shielding and bone resorption around total hip stems and the effects of flexible materials. Clinic Orthopaed Related Res. 124–134 1728998

[CR18] Krismer M, Biedermann R, Stöckl B, Fischer M, Bauer R, Haid C (1999) The prediction of failure of the stem in THR by measurement of early migration using EBRA-FCA. J Bone Joint Surg British 81(2):273–280. 10.1302/0301-620X.81B2.0810273 10.1302/0301-620x.81b2.8840 10204934

[CR19] Krstin N, Nackenhorst U, Lammering R (2000) Zur konstitutiven Beschreibung des anisotropen beanspruchungsadaptiven Knochenumbaus. Tech Mech 20(1):31–40

[CR20] Learmonth ID, Young C, Rorabeck C (2007) The operation of the century: total hip replacement. Lancet 370(9597):1508–1519. 10.1016/S0140-6736(07)60457-7 17964352 10.1016/S0140-6736(07)60457-7

[CR21] Lutz A (2011) Ein integrales Modellierungskonzept zur numerischen Simulation der Osseointegration und Langzeitstabilität von Endoprothesen. Ph. D. thesis, Gottfried Wilhelm Leibniz University, Hannover

[CR22] Lutz A, Nackenhorst U (2010) Numerical investigations on the biomechanical compatibility of hip-joint endoprostheses. Arch Appl Mech 80(5):503–512. 10.1007/s00419-009-0380-4

[CR23] Lutz A, Nackenhorst U (2012) Numerical investigations on the osseointegration of uncemented endoprostheses based on bio-active interface theory. Comput Mech 50:367–381. 10.1007/s00466-011-0635-0

[CR24] Nackenhorst U (1997) Numerical simulation of stress stimulated bone remodelling. Tech Mech 17(1):31–40

[CR25] Nackenhorst U (2018) Modeling of bone adaption processes. In: Altenbach H, Öchsner A (eds) Encyclopedia of Continuum Mechanics. Springer, Berlin Heidelberg, pp 1–11

[CR26] Ovesy M, Zysset PK (2023) Explicit non-linear finite element analysis for prediction of primary stability in uncemented total hip arthroplasty. In J. M. R. S. Tavares, C. Bourauel, L. Geris, and J. Vander Slote (Eds.), Computer Methods, Imaging and Visualization in Biomechanics and Biomedical Engineering II, Cham, pp. 128–142. Springer International Publishing.

[CR27] Pettersen SH, Wik TS, Skallerud B (2009) Subject specific finite element analysis of implant stability for a cementless femoral stem. Clin Biomech 24(6):480–487. 10.1016/j.clinbiomech.2009.03.009 10.1016/j.clinbiomech.2009.03.009 19368993

[CR28] Raffa ML, Nguyen VH, Haiat G (2019) Micromechanical modeling of the contact stiffness of an osseointegrated bone-implant interface. Biomed Eng Online 18(1):114. 10.1186/s12938-019-0733-3 31796076 10.1186/s12938-019-0733-3 PMC6889538

[CR29] Sun X, Curreli C, Viceconti M (2024) Finite element models to predict the risk of aseptic loosening in cementless femoral stems: a literature review. Appl Sci. 10.3390/app14083200

[CR30] Tarala M, Janssen D, Verdonschot N (2013) Toward a method to simulate the process of bone ingrowth in cementless THA using finite element method. Med Eng Phys 35(4):543–548. 10.1016/j.medengphy.2012.10.010 23195851 10.1016/j.medengphy.2012.10.010

[CR31] Taylor M, Prendergast PJ (2015) Four decades of finite element analysis of orthopaedic devices: Where are we now and what are the opportunities? J Biomech 48(5):767–778. 10.1016/j.jbiomech.2014.12.019 25560273 10.1016/j.jbiomech.2014.12.019

[CR32] Viceconti M, Muccini R, Bernakiewicz M, Baleani M, Cristofolini L (2000) Large-sliding contact elements accurately predict levels of bone-implant micromotion relevant to osseointegration. J Biomech 33(12):1611–1618. 10.1016/S0021-9290(00)00140-8 11006385 10.1016/s0021-9290(00)00140-8

[CR33] Viceconti M, Pancanti A, Dotti M, Traina F, Cristofolini L (2004) Effect of the initial implant fitting on the predicted secondary stability of a cementless stem. Med Biol Eng Comput 42:222–229. 10.1007/BF02344635 15125153 10.1007/BF02344635

[CR34] Webster D, Müller R (2011) In silico models of bone remodeling from macro to nano-from organ to cell. Wiley Interdiscip Rev Syst Biol Med 3(2):241–251. 10.1002/wsbm.115 20740496 10.1002/wsbm.115

[CR35] Weinans H, Huiskes R, Grootenboer H (1992) The behavior of adaptive bone-remodeling simulation models. J Biomech 25(12):1425–1441. 10.1016/0021-9290(92)90056-7 1491020 10.1016/0021-9290(92)90056-7

[CR36] Wolff J (1892) Das Gesetz der Transformation der Knochen. Hirschwald Verlag, Berlin

